# The ER-associated degradation adaptor SEL1L is dispensable for ER homeostasis and the differentiation of spermatogenic cells

**DOI:** 10.1016/j.jbc.2025.110283

**Published:** 2025-05-22

**Authors:** Nusrat Jahan Tushi, Zhibing Zhang, Shengyi Sun

**Affiliations:** 1 Department of Pharmacology, University of Virginia School of Medicine, Charlottesville, Virginia, USA; 2 Department of Physiology and Department of Obstetrics and Gynecology, Wayne State University School of Medicine, Detroit, Michigan, USA

**Keywords:** SEL1L, ERAD, sperm, spermatogenesis, spermiogenesis, endoplasmic reticulum, ER stress

## Abstract

The SEL1L-HRD1 complex is a critical component of the endoplasmic reticulum (ER)-associated protein degradation (ERAD) pathway, essential for maintaining ER homeostasis and cellular function. While the crucial roles of SEL1L and HRD1 in various physiological processes have been reported in mice and humans, their specific functions in male germ cells remain unexplored. Here, we show that, while SEL1L is highly expressed in spermatogenic cells, it is dispensable for their differentiation and ER homeostasis. SEL1L deletion in these cells does not affect sperm count, motility, male fertility, or testicular histology. Mechanistically, our data show that SEL1L loss reduces HRD1 protein levels in spermatids but unexpectedly, not in spermatocytes. Furthermore, SEL1L deficiency does not induce overt ER stress response, ER dilation, or cell death in the testes. Collectively, these findings indicate that SEL1L is not required for ER homeostasis or the differentiation of male germ cells.

The endoplasmic reticulum (ER)-associated degradation (ERAD) pathway plays a pivotal role in maintaining cellular homeostasis by identifying and degrading misfolded or damaged proteins from the ER, thereby ensuring proteostasis (1, 2). The suppressor/enhancer of Lin-12-like (SEL1L)-hydroxymethylglutaryl reductase degradation protein 1 (HRD1) complex is a central and highly conserved component of this pathway, crucial for regulating protein quality control across various tissues, including those involved in metabolism, immunity, tissue homeostasis, and hematopoietic cell differentiation (3, 4, 5, 6, 7, 8, 9, 10, 11, 12, 13, 14, 15, 16, 17, 18, 19, 20, 21, 22, 23, 24, 25, 26, 27). Notably, *in vivo*, SEL1L is essential for maintaining HRD1 stability and activity, especially in tissues such as the exocrine pancreas, liver, gut epithelium, and adipose tissues (12, 16, 21, 22, 23, 28). Despite the well-established importance of ERAD in cellular proteostasis, its specific role in male germ cells during spermatogenesis remains largely unexplored.

Spermatogenesis, a highly dynamic and coordinated process by which male germ cells differentiate into mature spermatozoa, is divided into three phases: (a) the mitosis phase when the spermatogonia self-renew and differentiate into spermatocytes, (b) the meiosis phase when spermatocytes undergo two meiotic divisions to form haploid round spermatids, and (c) the spermiogenesis phase when round spermatids elongate and differentiate into spermatozoa (29, 30). This complex process occurs in the seminiferous epithelium of the testicular tubules, where cohorts of differentiating germ cells progress toward the tubule lumen in a spatially and temporally synchronized manner (29, 30, 31). As a result, developing germ cells are organized into distinct, well-defined cellular associations known as the stages of the seminiferous epithelial cycle (30, 31). In mice, these stages can be categorized into 12 (I–XII) based on the dynamic morphological changes and specific cellular associations of differentiating germ cells (31).

Throughout spermatogenesis, ER proteostasis likely plays a critical role in the synthesis, folding, and quality control of various structural and signaling proteins essential for germ cell development (32, 33, 34, 35, 36, 37). Spermatocytes are highly active in RNA and protein synthesis, while round and elongated spermatids maintain tightly regulated transcription and translation to support the complex changes of spermiogenesis (38, 39). During spermiogenesis, the ER undergoes dramatic remodeling, collapsing from tubular networks into aggregates before disappearing in mature spermatozoa (40, 41). Despite previous studies, the regulation of ER homeostasis and the specific role of ERAD during spermatogenesis remain poorly understood. To explore the role of the SEL1L-HRD1 ERAD complex in this process, we investigated the role of SEL1L in male germ cells. Using a conditional *Sel1L* knockout model, we showed unexpectedly that SEL1L is dispensable for spermatogenesis and ER homeostasis in male germ cells.

## Results

### SEL1L and HRD1 proteins are highly expressed in spermatogenic cells

**Figure 1:**
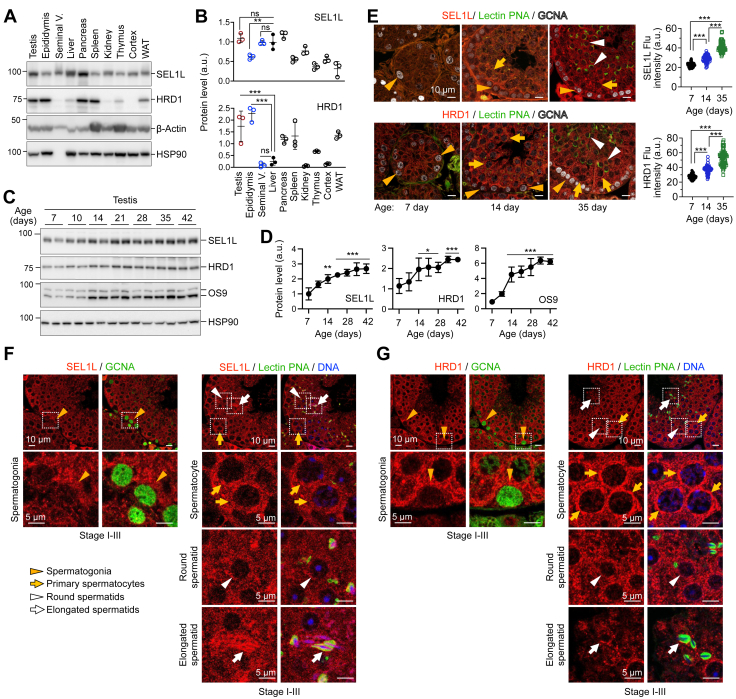
**SEL1L and HRD1 are expressed in spermatogenic cells.***A* and *B*, Western blot analysis of various tissues from 6-week-old wild-type mice, with quantitation of SEL1L and HRD1 shown in (*B*). With equal protein loading across samples, SEL1L and HRD1 quantification was not normalized due to the varying expression of housekeeping proteins β-actin and HSP90. N = 3. Values, mean ± SD. ∗∗, *p* < 0.01; ∗∗∗, *p* < 0.001; ns, not significant comparing different tissues to the liver by one-way ANOVA with Tukey multiple comparison test. *C* and *D*, Western blot analysis of testes from wild-type mice at different ages, with quantitation normalized to HSP90 shown in (*D*). N = 3. Values, mean ± SD. ∗, *p* < 0.05; ∗∗, *p* < 0.01; ∗∗∗, *p* < 0.001 comparing different ages to 7 days by one-way ANOVA with Tukey multiple comparison test. *E*–*G*, immunofluorescent staining of testes from wild-type mice at 7, 14, and 35 days old (*E*) and 6 weeks old (*F* and *G*), showing the expression of SEL1L and HRD1 in various cell types as illustrated. GCNA labels spermatogonia; lectin PNA labels acrosomes in round and elongated spermatids. In (*E*), the average immunofluorescent signal intensity of SEL1L and HRD1 in individual seminiferous tubules was quantitated. 60 to 75 seminiferous tubules from three individual mice were quantitated at each age. Values, mean ± SD. ∗∗∗, *p* < 0.001 by one-way ANOVA with Tukey multiple comparison test. In (*F* and *G*), insets of higher magnification are shown below. Representative data from n = 3 in (*E*) and n = 4 in (*F* and *G*).

Both SEL1L and HRD1 proteins were abundantly expressed in the testis and other male reproductive organs, such as the epididymis and seminal vesicle, in adult mice (Fig. 1, *A* and *B*). To understand the expression of SEL1L and HRD1 proteins at different phases of spermatogenesis, we examined developing testes during postnatal Days 7 to 42, representing the first wave of spermatogenesis in mice (30, 42): at postnatal Day 7 with only spermatogonia and somatic Sertoli cells, at day 10 to 14 when meiosis begins with the accumulation of leptotene and large pachytene spermatocytes, and beyond Day 21 when round spermatids are generated and undergo elongation and differentiation (Fig. S1*A*). Both SEL1L and HRD1 protein levels increased with age in the testis during the first wave of spermatogenesis (Fig. 1, *C* and *D*). Other ER proteins, such as ER lectin OS9, inositol-requiring enzyme 1 (IRE1α), binding immunoglobulin protein (BiP), and glucose-regulated protein 94 (GRP94), exhibited similar protein expression patterns (Figs. 1, *C* and *D*, and S1, *B* and *C*). Immunofluorescent staining and confocal microscopy further confirmed that SEL1L and HRD1 protein levels in the testis increased with age between 7 and 35 days postnatally, with a high expression in spermatocytes at 14 days, and in both spermatocytes and spermatids at 35 days (Figs. 1*E* and S1*D*). In adult mice, SEL1L and HRD1 proteins were expressed in GCNA^+^ spermatogonia, spermatocytes, and lectin-PNA^+^ acrosome-containing round and elongated spermatids (Figs. 1, *F* and *G* and S1, *E* and *F*). Similarly, the ER chaperone GRP94 exhibited a comparable expression pattern across these cell types (Fig. S1*G*). Together, these results demonstrate that SEL1L-HRD1 ERAD is induced during testis development and is expressed in spermatogenic cells, indicating potential physiological functions during spermatogenesis.

### Generation of male germ cell-specific *Sel1L* knockout mice

**Figure 2:**
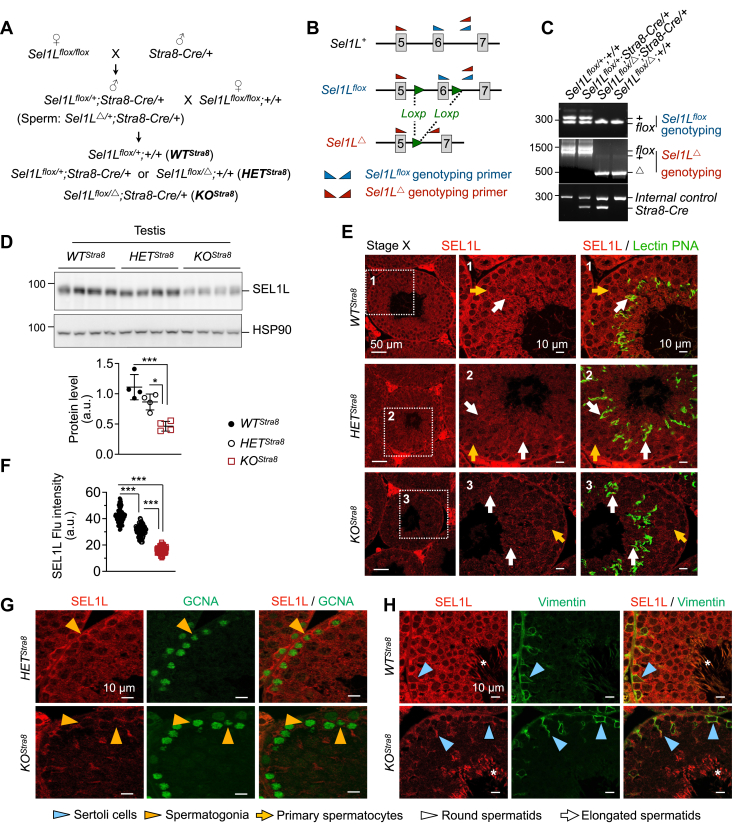
**Generation of male germ cell-specific *Sel1L* knockout mice.***A*, diagram illustrating the generation of male germ cell-specific *Sel1L* knockout mice (*KO*^*Stra8*^), wildtype (*WT*^*Stra8*^), and heterozygous (*HET*^*Stra8*^) littermates. *B*, diagram of the *Sel1L* locus with primers for genotyping illustrated. *C*, representative genotyping results. *D*, Western blot analysis of testes from 6-week-old *WT*^*Stra8*^, *HET*^*Stra8*^, and *KO*^*Stra8*^ littermates, with quantitation of SEL1L normalized to HSP90 shown below. N = 4 per cohort. *E*–*H*, immunofluorescent staining of testes from 6-week-old *WT*^*Stra8*^, *HET*^*Stra8*^, and *KO*^*Stra8*^ littermates, showing the ablation of SEL1L in spermatocytes and spermatids (*E*), but not in GCNA^+^ spermatogonia (*G*), or vimentin^+^ Sertoli cells (*H*). Insets of higher magnification are shown on the *right* in (*E*). The immunofluorescent signal intensity of SEL1L in individual seminiferous tubules is quantified in (*F*). In (*H*), ∗ indicates non-specific staining by the SEL1L antibody in spermatid flagella at stage VII-VIII. Values, mean ± SD. ∗, *p* < 0.05; ∗∗∗, *p* < 0.001 by one-way ANOVA with Tukey multiple comparison tests. Representative data from n = 4 mice per cohort.

To investigate the role of SEL1L-HRD1 ERAD in male germ cells, we generated male germ cell-specific *Sel1L* knockout mice (*Sel1L*^*flox/Δ*^*; Stra8-Cre*, and *KO*^*Stra8*^) by crossing *Sel1L*^*flox/flox*^ females with transgenic male mice with an optimized variant of Cre recombinase under the control of a male germ cell-specific promoter *Stra8* (*Stra8-Cre*) (43) (Fig. 2*A*). The resulting mice were genotyped by primers to detect *Sel1L*^*flox*^, *Sel1L*^*Δ*^ (*Sel1L* deletion by spermatogenic *Stra8-Cre* expression), and *Stra8-Cre* alleles (Fig. 2, *B* and *C*). Western blot showed that SEL1L protein levels were markedly reduced by 60% in the testes of *KO*^*Stra8*^ mice compared to wildtype (*Sel1L*^*flox/+*^*, WT*^*Stra8*^) littermates, while *Sel1L* heterozygous mice (*Sel1L*^*flox/+*^*;Stra8-Cre* or *Sel1L*^*flox/Δ*^, combined as *HET*^*Stra8*^) exhibited an intermediate expression level (Fig. 2*D*). Immunofluorescent staining revealed the loss of SEL1L expression in spermatocytes, round, and elongated spermatids of *KO*^*Stra8*^ mice (Figs. 2, *E* and *F* and S2). In contrast, SEL1L expression was still observed in GCNA^+^ spermatogonia of *KO*^*Stra8*^ mice (Fig. 2*G*), in line with a previous report showing only a partial activity of *Stra8*-dirven Cre in spermatogonia (43). SEL1L expression was not affected in vimentin^+^ Sertoli cells in *KO*^*Stra8*^ mice (Figs. 2*H* and S1*D*). Together, we generated male germ cell-specific *Sel1L* knockout mice with SEL1L deficiency in spermatogenic cells.

### HRD1 is depleted in spermatids, but not spermatocytes, in *KO*^*Stra8*^ mice

**Figure 3:**
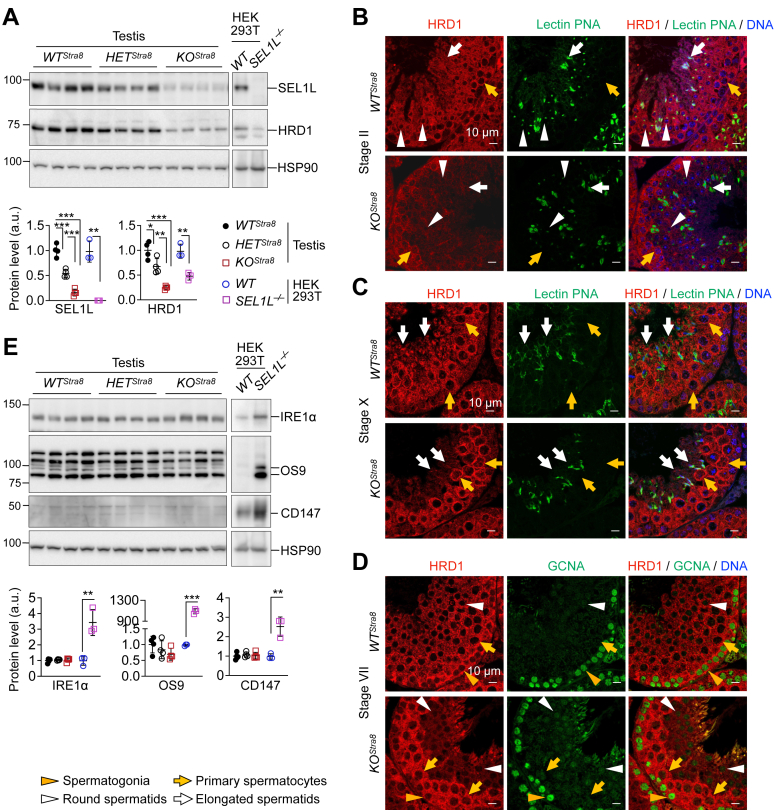
***Sel1L* ablation leads to the loss of HRD1 in spermatids, but not spermatocytes.***A*, Western blot analysis of testes from 6-week-old *WT*^*Stra8*^, *HET*^*Stra8*^, and *KO*^*Stra8*^ littermates, with quantitation of SEL1L and HRD1 normalized to HSP90 shown below. *WT* and *SEL1L*^*−/−*^ HEK293T cells were shown as controls. *B*–*D*, immunofluorescent staining of testes from 6-week-old *WT*^*Stra8*^ and *KO*^*Stra8*^ littermates, showing HRD1 expression in spermatocytes and its absence in round and elongated spermatids. Lectin PNA labels acrosomes in round and elongated spermatids; GCNA labels spermatogonia. Representative data from n = 4 mice per cohort. *E*, Western blot analysis of ERAD substrate proteins in testes from 6-week-old *WT*^*Stra8*^, *HET*^*Stra8*^, and *KO*^*Stra8*^ littermates, with quantitation normalized to HSP90 shown below. Note the accumulation of ERAD substrates in *SEL1L*^*−/−*^ HEK293T cells but not *KO*^*Stra8*^ testes. In (*A* and *E*), N = 4 for testes, n = 3 for HEK293T cells. Values, mean ± SD. ∗, *p* < 0.05; ∗∗, *p* < 0.01; ∗∗∗, *p* < 0.001 by one-way ANOVA with Tukey multiple comparison tests.

We next investigated whether SEL1L is required for HRD1 expression in spermatogenic cells. In line with many other tissues (12, 14, 15, 16, 23, 25, 44, 45), Western blot analysis of total testis lysates showed that the loss of SEL1L led to reduced HRD1 protein levels by about 80% in the testes of both 7-week- and 8-month-old mice (Figs. 3*A* and S3*A*). Immunofluorescent staining revealed that, in Stage II seminiferous tubules, HRD1 protein was diminished in Step 2 round spermatids (white arrowheads) and Step 14 elongated spermatids (white arrows, Fig. 3*B*) of *KO*^*Stra8*^ mice compared to *WT*^*Stra8*^ controls. However, despite SEL1L being clearly absent in all stages of spermatogenesis in *KO*^*Stra8*^ mice (Figs. 2*E* and S2), HRD1 protein level was unaffected in pachytene spermatocytes of *KO*^*Stra8*^ mice (yellow arrows, Fig. 3*B*). Consistent observations were made in seminiferous tubules at stages VII and X, where spermatocytes were more abundant compared to those in Stage II (Fig. 3, *C* and *D*). We then examined ERAD activity in male germ cells by performing Western blot analyses of known ERAD substrates, IRE1α, OS9, and CD147 (12, 23, 46, 47) in testes of both young and old *KO*^*Stra8*^ mice. Surprisingly, the loss of SEL1L did not affect the protein levels of IRE1α, OS9, or CD147 proteins in the testes of *KO*^*Stra8*^ mice (Fig. 3*E* and S3*B*). In contrast, all three proteins were increased in *SEL1L*-deficient HEK293T cells (Fig. 3, *A* and *E*). Hence, these data suggest that SEL1L is dispensable for ERAD function in spermatogenic cells and is required for HRD1 protein stability in spermatids, but not in spermatocytes. The underlying mechanism remains unclear.

### Male germ cell-specific *Sel1L* ablation does not affect fertility

**Figure 4:**
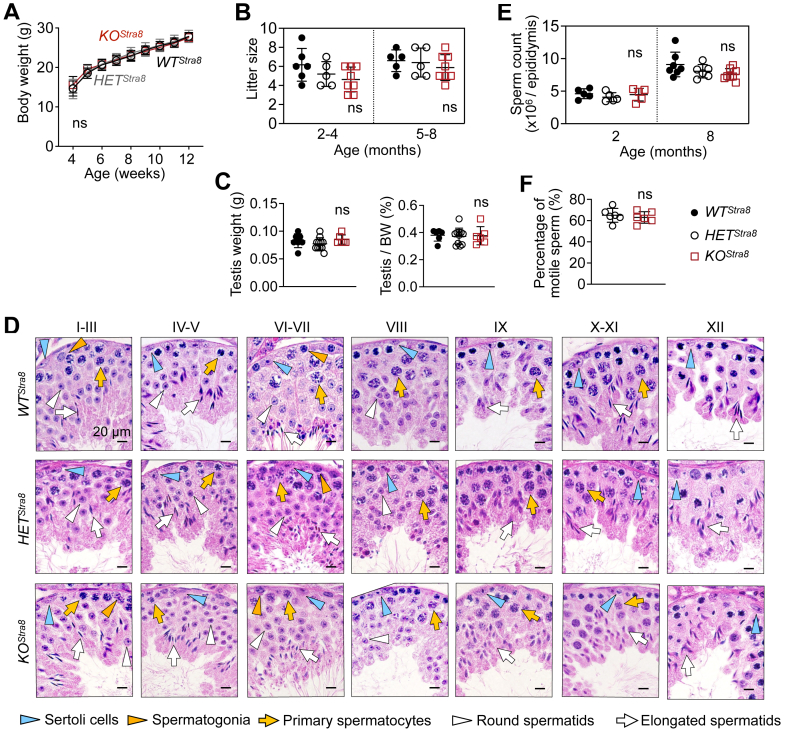
***Sel1L* deficiency in male germ cells does not affect fertility.***A*, growth curve of *WT*^*Stra8*^*, HET*^*Stra8*^, and *KO*^*Stra8*^ littermates. N = 8 per cohort. *B*, breeding test mating *WT*^*Stra8*^*, HET*^*Stra8*^, and *KO*^*Stra8*^ males of varying ages with 2- to 4-month-old wild-type females. The number of pups born per litter is shown. N = 4 for *WT*^*Stra8*^ and *HET*^*Stra8*^, n = 5 for *KO*^*Stra8*^. *C*, weight and normalized weight of testes from 6-week-old cohorts. N = 6 to 11. *D*, representative images of H&E stained testis sections from 6-week-old *WT*^*Stra8*^*, HET*^*Stra8*^, and *KO*^*Stra8*^ littermates, showing various cell types at different stages of the seminiferous epithelium cycle. Representative images from n = 4 mice per cohort. *E* and *F*, mature sperm count (*E*) and percentage of motile sperm (*F*) released from epididymis of *WT*^*Stra8*^*, HET*^*Stra8*^, and *KO*^*Stra8*^ littermates. N = 5 for 2-month cohorts, and n = 6 to 8 for 8-month cohorts in (*E*); n = 6 in (*F*). Values, mean ± SD. Ns, not significant by one-way ANOVA with Tukey multiple comparison tests in (*B*, *C*, and *E*), and by two-tailed Student’s *t* test in (*F*).

*KO*^*Stra8*^ mice showed comparable growth to their *WT*^*Stra8*^ and *HET*^*Stra8*^ littermates, with no signs of physical abnormalities (Fig. 4*A*). To assess fertility, *WT*^*Stra8*^, *HET*^*Stra8*^, and *KO*^*Stra8*^ males of varying ages were bred with 2- to 4-month-old wildtype females. Fertility was comparable across *WT*^*Stra8*^, *HET*^*Stra8*^, and *KO*^*Stra8*^ males with normal-sized litters sired from 2- to 8-month-old of age (Fig. 4*B*). Testes weighed similarly among all three cohorts (Fig. 4*C*). Histological examination of different stages of the seminiferous epithelial cycle failed to generate notable differences between *KO*^*Stra8*^ and other control littermates (Fig. 4*D*). Cauda epididymis sperm number and motility, as well as the weight and morphology of epididymis and seminal vesicle, were not affected by male germ cell SEL1L deficiency either (Figs. 4, *E* and *F* and S4).

### Male germ cell-specific *Sel1L* ablation does not affect spermatogenesis

**Figure 5:**
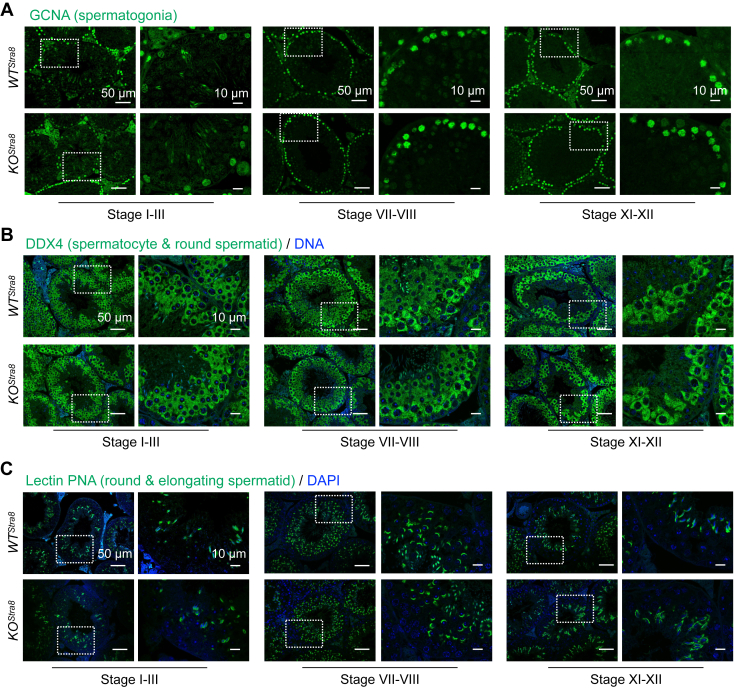
**Spermatogenesis is not affected by male germ cell-specific *Sel1L* ablation.** Immunofluorescent staining of testes from 6-week-old *WT*^*Stra8*^ and *KO*^*Stra8*^ littermates, showing (*A*) GCNA, a germ-cell specific marker. *B*, DDX4, a spermatocyte and round spermatid-specific marker. *C*, lectin PNA, a sperm acrosome marker at different stages of the seminiferous epithelium cycle. Insets of higher magnification shown on the *right*. Representative images from n = 4 mice per cohort.

We next investigated whether SEL1L deficiency affects spermatogenesis by analyzing spermatogenic cells at specific differentiation stages using immunofluorescent staining. Throughout the seminiferous epithelial cycle, the number and distribution of GCNA^+^ spermatogonia (Fig. 5*A*), DDX4^+^ spermatocytes and round spermatids (Fig. 5*B*), and lectin-PNA^+^ round and elongated spermatids (Fig. 5*C*) were comparable between *WT*^*Stra8*^ and *KO*^*Stra8*^ cohorts. Taken together, these data suggest that SEL1L in male germ cells is dispensable for fertility and spermatogenesis.

### *Sel1L* ablation does not affect ER homeostasis in spermatogenic cells

**Figure 6:**
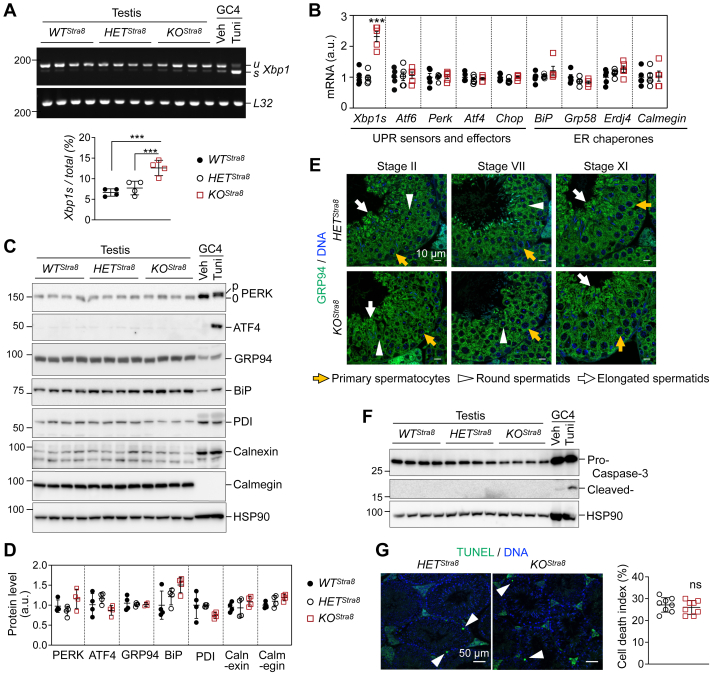
**ER homeostasis is not affected by male germ cell-specific *Sel1L* ablation.***A* and *B*, RT-PCR analysis of *Xbp1* splicing (*A*) and qPCR analysis (*B*) in testes from 6-week-old *WT*^*Stra8*^*, HET*^*Stra8*^, and *KO*^*Stra8*^ littermates. In (*A*), *u* and *s* denote unspliced and spliced *Xbp1* mRNA, respectively. Percentage of *Xbp1* splicing is quantitated below the images. Male germ GC-4 cells treated with 2 μg/ml tunicamycin (Tuni) for 4 h serve as a positive control. N = 4 in A, n = 5 in B. *C* and *D*, Western blot analysis of testes from 6-week-old *WT*^*Stra8*^*, HET*^*Stra8*^, and *KO*^*Stra8*^ littermates, with quantitation normalized to HSP90 shown in (*D*). GC-4 cells treated with 2 μg/ml tunicamycin for 4 h serve as a positive control. N = 4 per cohort. *E*, immunofluorescent staining of GRP94 in testis sections from 6-week-old *HET*^*Stra8*^ and *KO*^*Stra8*^ littermates. Representative images from n = 4 per cohort. *F*, Western blot analysis of testes from 6-week-old *WT*^*Stra8*^, *HET*^*Stra8*^, and *KO*^*Stra8*^ littermates. GC-4 cells treated with 2 μg/ml tunicamycin for 24 h serve as a positive control. *G*, TUNEL staining (*green*) with nuclei stained with DAPI in testes from 6-week-old *HET*^*Stra8*^ and *KO*^*Stra8*^ littermates. *White* arrowheads, TUNEL-positive cells. The cell death index represents the percentage of seminiferous tubules containing at least one apoptotic cell, quantified from low-magnification whole testis sections of n = 8 mice per cohort. Values, mean ± SD. ∗∗∗, *p* < 0.001; ns or not labeled, not significant by one-way ANOVA with Tukey multiple comparison tests in (*A*, *B* and *D*), and by two-tailed Student’s *t* test in (*G*).

The dispensable role of SEL1L in male fertility and spermatogenesis prompted us to test mechanistically whether and how SEL1L deficiency affects ER homeostasis in male germ cells. We first examined the activation of two unfolded protein response (UPR) pathways, initiated by UPR sensors IRE1α and PKR-like ER kinase (PERK). There was a very mild 2-fold increase of X box binding protein 1 (*Xbp1*) mRNA splicing in *KO*^*Stra8*^ testes compared to *WT*^*Stra8*^ and *HET*^*Stra8*^ controls, indicating a minor activation of the IRE1α pathway (Figs. 6, *A* and *B* and S5*A*). Nonetheless, the expression of ER chaperones, including *BiP*, glucose-regulated protein 58 (*Grp58*), endoplasmic reticulum DNA J domain-containing protein 4 (*Erdj4*), and *calmegin*, as well as key UPR sensors and effectors such as *Perk,* activating transcription factor 4 (*Atf6*), activating transcription factor 4 (*Atf4*), and C/EBP homologous protein (*Chop*), was unchanged in both young and old *KO*^*Stra8*^ testes compared to *WT*^*Stra8*^ and *HET*^*Stra8*^ controls (Figs. 6*B* and S5*A*). On the protein level, the total protein and phosphorylation of UPR sensor PERK were unaffected in *KO*^*Stra8*^ testes, as were its downstream effector ATF4 (Figs. 6, *C* and *D* and S5*B*). Protein levels of ER chaperones such as GRP94, BiP, protein disulfide isomerase (PDI), calnexin, and calmegin showed no significant changes in *KO*^*Stra8*^ testes (Figs. 6, *C* and *D* and S5*B*). Immunofluorescent staining revealed comparable levels of ER chaperone GRP94 in different spermatogenic cells of *HET*^*Stra8*^ and *KO*^*Stra8*^ mice (Figs. 6*E* and S5*C*). Furthermore, there was no increase in cell death in *KO*^*Stra8*^ testes as demonstrated by caspase-3 cleavage and TUNEL staining (Fig. 6, *F* and *G*). As a positive control, male germ GC4 cells treated with an ER stress inducer, tunicamycin did exhibit a strong UPR response and cell death (Fig. 6, *A* and *C*, and *F*). These data suggest a dispensable role of SEL1L in ER homeostasis during spermatogenesis.

**Figure 7:**
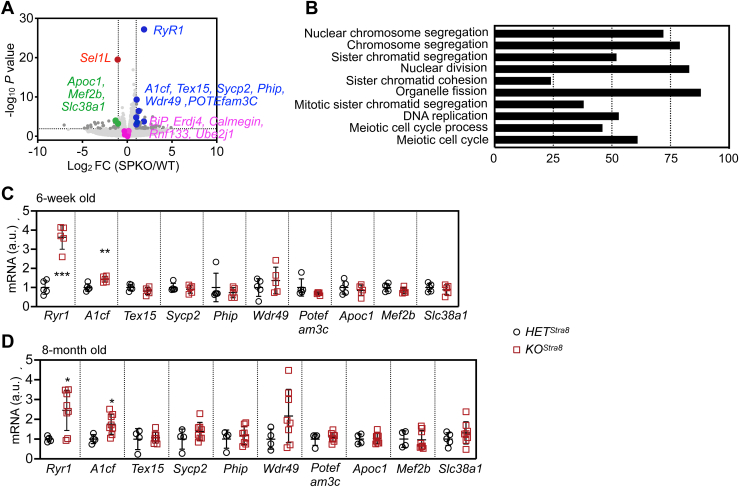
**Transcriptomics analysis reveals normal ER homeostasis with the loss of SEL1L in male germ cells.***A*, volcano plot of whole genome RNA sequencing analysis in testes from 7-week-old *HET*^*Stra8*^ and *KO*^*Stra8*^ littermates. X and Y axes represent log2 fold change and -log10 *p*-value, respectively. The horizontal and vertical dotted lines represent two fold change and an adjusted *p*-value <0.01, respectively. *B*, pathway analysis showing the differentially regulated pathways. *C* and *D*, qPCR analysis of testes from 7-week-old and 8-month-old *HET*^*Stra8*^ and *KO*^*Stra8*^ littermates, showing significantly upregulated or downregulated genes comparing *KO*^*Stra8*^ to *HET*^*Stra8*^ littermates identified in RNA sequencing analysis. Values, mean ± SD. ∗, *p* < 0.05; ∗∗, *p* < 0.01; ∗∗∗, *p* < 0.001 by two-tailed Student’s *t* test.

We then performed an unbiased whole-genome RNA sequencing analysis of the testes from 7-week-old *HET*^*Stra8*^ and *KO*^*Stra8*^ mice. In total, 69 genes were upregulated by over twofolds and 28 genes were downregulated by over twofolds in the testes of *KO*^*Stra8*^ mice compared to *HET*^*Stra8*^ littermates, with adjusted *p* value <0.01 (Fig. 7*A* and Table S1). Consistent with our earlier analysis, the transcripts of ER chaperones *BiP*, *Erdj4*, and *calmegin* were not affected by the loss of SEL1L in the testis, while *Sel1L* mRNA was significantly reduced (Fig. 7*A*). In addition, the expression of two ERAD-related genes, *Ube2j1* and *Rnf133*, previous implicated in spermatogenesis (48, 49), remained unchanged in the absence of SEL1L (Fig. 7*A*). Indeed, pathway analysis showed that UPR was not among the pathways that were differentially regulated (Fig. 7*B*). Quantitative PCR analysis of testes from either 7-week- or 8-month-old mice showed a consistent induction of ER calcium channel ryanodine receptor 1 (*Ryr1*), which regulates spermatogonia self-renewal and differentiation (50), and RNA-editing protein APOBEC1 complementation factor (*A1cf*), which regulates testicular germ-cell differentiation and proliferation (51), by 2 to 3 folds in the *KO*^*Stra8*^ testes compared to *HET*^*Stra8*^ controls (Fig. 7, *C* and *D*). Taken together, the global transcriptomics analysis demonstrates a lack of UPR to the loss of SEL1L in the testis, thus suggesting a dispensable role of SEL1L in the maintenance of ER homeostasis in male germ cells.

### SEL1L deficiency does not alter ER ultrastructure in spermatogenic cells

**Figure 8:**
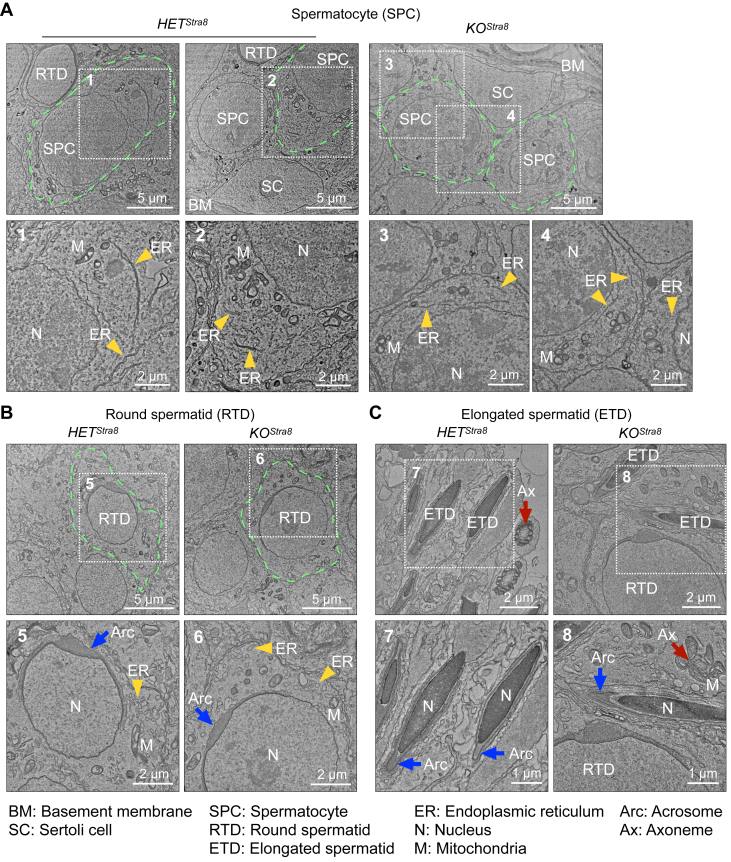
***Sel1L* deficiency does not affect the ultrastructure of male germ cells.** Transmission electron microscopy (TEM) images of testes from 6-week-old *HET*^*Stra8*^ and *KO*^*Stra8*^ littermates, showing primary spermatocytes with large, rounded nuclei (*A*), round spermatid with cap-shaped acrosome (*B*), and elongated spermatids with condensed chromatin and elongated acrosome (*C*). *Green dotted lines* illustrate individual cells, and insets of higher magnification are numbered and shown below. *Yellow arrowheads*, ER; *blue arrows*, acrosome; *red arrows*, axoneme. N = 2 per cohort.

Finally, we performed transmission electron microscopy (TEM) to delineate the impact of SEL1L deficiency on organellar structure in different spermatogenic cells. The ER in spermatocytes was observed as flat sheets, concentrically arranged around the nucleus (yellow arrowheads, Fig. 8*A*). Comparing *HET*^*Stra8*^ and *KO*^*Stra8*^ mice, no differences in the morphology or abundance of ER in spermatocytes were observed (Fig. 8*A*). Further into the seminiferous tubules, round spermatids with round nuclei and acrosome caps, as well as elongated spermatids with condensed nuclei and overlaying acrosomes were abundantly found (Fig. 8, *B* and *C*). In both cell types, the loss of SEL1L did not affect the morphology or distribution of ER sacs and mitochondria in round spermatids (yellow arrowheads and “M”, Fig. 8*B*), nor the formation of acrosomes and axonemes in round and elongated spermatids (blue and red arrows, Fig. 8, *B* and *C*). In addition, we did not observe any abnormal retention of ER or cytoplasm in *KO*^*Stra8*^ elongated spermatids, suggesting normal cytoplasm removal, in contrast to the defects reported in mice deficient in ubiquitin conjugating enzyme E2 J1 (UBE2J1), a key E2 enzyme in the ERAD pathway (48). Taken together, we conclude that SEL1L-HRD1 ERAD is dispensable for the dynamic reorganization of organelles during spermiogenesis.

## Discussion

In this study, we demonstrate that SEL1L is dispensable for spermatogenesis in male germ cells. Deletion of *Sel1L* does not affect spermatogenesis or male fertility. Additionally, SEL1L deficiency reduces HRD1 protein levels in spermatids but not in spermatocytes. To our knowledge, this is the first *in vivo* evidence showing that SEL1L loss does not cause overt abnormalities under physiological conditions. However, its role under pathological or stress conditions, such as those associated with elevated ER stress (52, 53, 54, 55, 56, 57, 58, 59) warrants further investigation.

During spermiogenesis, the final phase of spermatogenesis, the ER undergoes dramatic morphological changes, degenerating from branched tubular networks to collapsed aggregates and eventually disappearing in mature spermatozoa (40, 41). Indeed, previous studies have implicated ERAD in this process (48, 49). The loss of ubiquitin conjugating enzyme E2 J1 (UBE2J1), a key E2 enzyme in the ERAD pathway, causes male infertility and defective ER and cytoplasm removal during spermiogenesis in mice (48). UBE2J1 is well-known to function with the SEL1L-HRD1 E3 ligase complex to mediate ERAD functions (9). However, in light of the findings in this study, we propose that other E3 ligases may work with UBE2J1 to allow ER degeneration. Indeed, another recent report suggests that RNF133, a relatively unknown putative ER-localized E3 ligase, may interact with UBE2J1 and mediate ER degeneration in spermiogenesis (49). Nevertheless, as RNF133 deficiency only leads to subfertility while UBE2J1 deficiency leads to total infertility, additional factors may play a role in this process. The functional redundancy and compensation among the different ERAD machineries during spermiogenesis are interesting open questions. In addition, the role of HRD1 in this process requires the generation and characterization of male germ cell-specific *Hrd1* knockout mice.

For germ cells during spermatogenesis, pachytene spermatocytes are the largest cell type with highly active RNA and protein synthesis in preparation for the next phases (38). In round and elongated spermatids, although tightly regulated, transcription, and translation of specific proteins still occur to ensure the complex morphological and biochemical modifications during spermiogenesis (39). Despite a high abundance of SEL1L and HRD1 proteins in pachytene spermatocytes as well as in round and elongated spermatids, the loss of SEL1L in spermatocytes and spermatids did not affect spermatogenesis. Indeed, there was little to no induction of UPR and ER chaperones, and no ER dilation in the absence of SEL1L, suggesting that SEL1L is dispensable during spermatogenesis. Future studies are required to address many unanswered questions, such as the nature of alternative ERAD machineries in germ cells during spermatogenesis, the role of ERAD in ER degeneration and cytoplasm removal during spermiogenesis, and whether HRD1 in spermatocytes is functional in the absence of SEL1L.

The male germ cell-specific *Sel1L* knockout mice were generated using transgenic mice expressing *Stra8* promoter-driven Cre recombinase (43), which did not deplete SEL1L in spermatogonia (Fig. 2*G*). It remains unclear whether SEL1L-HRD1 ERAD is required for the self-renewal and differentiation of these germline stem cells. Careful future investigations are needed to further advance our understanding of ERAD and ER quality control in germ cells and their implications for fertility.

## Experimental procedures

### Sex as a biological variable

Our study exclusively examined male mice because it investigates spermatogenesis.

### Generation of germ cell-specific *SEL1L*^*Stra8*^ knockout mice

Male germ cell specific *Sel1L* knockout mice were generated by breeding *Sel1L*^*flox/flox*^ females (12) with male mice expressing germ cell-specific *Stra8* promoter driven optimized variant of Cre recombinase (*Stra8-Cre*, Jax #008208) (43). Genotyping primers are listed below:

*Stra8-Cre*: forward: 5′-AGATGCCAGGACATCAGGAACCTG-3′; and reverse: 5′-ATCAGCCACACCAGACACAGAGATC-3′;

*Sel1L*^*flox*^: forward: 5′-CTGACTGAGGAAGGGTCTC-3′; and reverse: 5′-GCTAAAAACATTACAAAGGGGCA-3′;

*Sel1L*^*Δ*^: forward: 5′-GAAGCAATAGGAAGAGCCAAAA-3′; and reverse: 5′-GCTAAAAACATTACAAAGGGGCA-3′.

Mice were fed a standard rodent chow diet and housed in a temperature-controlled environment with 12-h light/dark cycles with free access to food and water. Mice were euthanized by cervical dislocation under isoflurane-induced anesthesia. Tissues were collected and immediately either fixed for histology analysis or frozen in liquid nitrogen.

### Assessment of fertility, spermatozoa counting, and motility assay

For the breeding test, each 2-month-old male was housed with one wild-type female continuously for 6 months. Cages are checked and recorded daily for pregnancy and pups per delivery. For spermatozoa counting and motility assay, the cauda epididymis was collected, incised, and incubated at 37 °C in 1 ml warm PBS for 10 min to release the sperm. The sperm numbers were counted using a hemocytometer chamber. Sperm motility assay was performed as previously described (60) using a Nikon TE200E inverted microscope with SANYO color CCD, Hi-resolution camera 9VCC-3972. Movies were recorded using Pinnacle Studio HD (Ver. 14.0) software for 10s/field. To calculate the percentage of motile sperm, a total of five fields was randomly observed using ImageJ (National Institutes of Health) for each mouse.

### Western blot and quantitation

Preparation of cell and tissue lysates and Western blotting was performed as previously described (23). The following antibodies were used in this study: SEL1L (Abclonal customized) (61), HRD1 (Abclonal customized E15102, used for mouse tissues) (62), HRD1 (proteintech 13473-1-AP, used for HEK293T cell), OS9 (Abcam ab109510), IRE1α (CST 14C10), BiP (CST C50B12), GRP94 (Novus NBP2-42379), calmegin (Proteintech 12629-1-AP), PERK (CST 3192), ATF4 (CST 11815), calnexin (Enzo ADI-SPA-860-D), PDI (Enzo ADI-SPA-890), CD147 (Proteintech 11989-1-AP), caspase-3 (CST 9662), and HSP90 (Santa Cruz sc7947). Antibody specificity for Western blot was validated by the manufacturers or in previous reports (61, 62). Secondary antibodies, goat anti-rabbit IgG HRP (Bio-rad), were used at 1:6000 dilution. Western blot membranes were developed using the Clarity Western ECL Substrate (Bio-rad), and the signal was detected with a ChemiDOC imager (Bio-rad). Image LAB software was used to quantify the band density.

### Transmission electron microscopy (TEM)

Mice were anesthetized and perfused with perfusion fixation buffer containing 2.5% glutaraldehyde, 2% formaldehyde in 0.1 M sodium cacodylate buffer (Electron Microscopy Sciences). Testis tissues were collected, cut into small pieces (about 1–2 mm cubes), and incubated in primary fixation buffer containing 3% glutaraldehyde, 3% formaldehyde in 0.1M sodium cacodylate overnight at 4  °C. The tissues were then submitted to the University of Michigan Microscopy Core for washing, embedding and sectioning at 70 nm. The grid containing tissue sections was imaged by a Talos F200X transmission electron microscope at Wayne State University Lumigen Instrument Center.

### RNA extraction, RT-PCR, and quantitative real-time PCR

Testis RNA was isolated as previously described (63). Briefly, total RNA was extracted from the testis using RNA-Stat 60 (IsoTex Diagnostics), chloroform, and precipitated by isopropanol. RNA quality was determined by measuring the OD260/280 and visualizing it on an agarose gel. cDNA was generated using the Superscript III reverse transcriptase (Thermofisher). RT-PCR for *Xbp1* splicing was performed as previously described (64) using GoTaq PCR Master Mixes (Promega). Quantitative PCR (qPCR) was performed using 2X Universal SYBR Green Fast qPCR Mix (Abclonal). *U36B4* was used as the reference. RT-PCR and qPCR primer sequences are listed below:

#### For RT-PCR

mouse *Xbp1* (ACACGCTTGGGAATGGACAC, CCATGGGAAGATGTTCTGGG), mouse *L32* (GAGCAACAAGAAAACCAAGCA, TGCACACAAGCCATCTACTCA).

#### For qPCR

mouse *BiP* (CAAGGATTGAAATTGAGTCCTTCTT, GGTCCATGTTCAGCTCTTCAAA), mouse *Xbp1s* (GAGTCCGCAGCAGGTG, GTGTCAGAGTCCATGGGA), mouse *Chop* (CCAGAAGGAAGTGCATCTTCA, ACTGCACGTGGACCAGGTT), mouse *Atf6* (TACCACCCACAACAAGACCA, TGATGATCCCGGAGATAAGG)

mouse *Atf4* (CGAGATGAGCTTCCTGAACAGC, GGAAAAGGCATCCTCCTTGC)

mouse *Perk* (TCAAGTTTCCTCTACTGTTCACTCA, CGGGAAACTCCAAGTTCTCA)

mouse *Grp58* (GAGGCTTGCCCCTGAGTATG, GTTGGCAGTGCAATCCACC)

mouse *Erdj4* (CAGAATTAATCCTGGCCTCC, ACTATTGGCATCCGAGAGTG)

mouse *Calmegin* (AGAGGCAGAGGAGGAGAAGG, CTTCTCTTCCGGTTCACCAA)

mouse *Ryr1* (TACTTCGACACAACCCCACA, AGTGTGCTCTGTCTCATCCT)

mouse *Wdr49* (CACGTTTGGGTCCTTATGCC, GAAGTTCTTTGGGGAATAGGCT)

mouse *Phip* (AAGTCAGCCGCAGTCATAGA, CTCCCAGGTCCTCTCTTCAC)

mouse *Tex15* (ACAGACAGTATCCAAACATCCA, TTGGTGTGCAAGTGTCTTGT)

mouse *A1cf* (CACTTCTGCCACTGTGTTCC, GGTTCCATATGCATCGCCTC)

mouse *Sycp2* (ATGACCTCTCAAGCTCAGGG, CCAAGTCTCGCAAAGTCTGT)

mouse *Apoc1* (TCCGGAACATTGGAGAGCAT, GCCAAATGCCTCTGAGAACC)

mouse *Slc38a1* (GCTGGCCAAGAAGACAAAGT, CCGTCCTGGTTCGTGATTTT)

mouse *Mef2B* (CTGCAACCCTCACCCTGG, AGACTTGATGCTGACCGGAG)

mouse *Potefam3C* (GTCACCCAGTCCCACAGTCT, TTCTCTGAGCTGACGATCCA)

mouse *U36B4* (CGTCCTCGTTGGAGTGACA, CGGTGCGTCAGGGATTG).

### Histology, immunofluorescence, and TUNEL staining

Testes were fixed in a fixation buffer containing 11.1% formalin, 5% glacial acetic acid, and 15% ethanol overnight at 4 °C and processed by the University of Michigan In-Vivo Animal Core on a fee-for-service basis. The H&E slides were imaged using an Aperio Scanscope (Leica). For immunofluorescent staining, paraffin sections were rehydrated and boiled for antigen retrieval in sodium citrate buffer (18mM citric acid and 9.35 mM sodium citrate, pH 6.0) for 20 min, followed by blocking (5% normal donkey serum, 0.5% Tween-20 in PBS) at room temperature for 1 h. The slides were incubated with primary antibodies at 4 °C overnight, followed by secondary antibodies at room temperature for 1 h using Alexa fluor 488 or 594 affinipure donkey anti-mouse or rabbit IgG (H + L) (Jackson ImmunoResearch). For negative controls, normal serum of the host animals of the respective primary antibodies was used in place of the primary antibody, followed by the same secondary antibody incubation. All antibodies were diluted in PBS containing 5% normal donkey serum and 0.5% Tween-20. The nuclei and slides were stained and mounted by Prolong Diamond Antifade Reagent with DAPI (Invitrogen P36971). The following antibodies were used in this study: SEL1L (Abcam ab78298), HRD1 (Abclonal customized E15102), GRP94 (Novus NBP2-42379), DDX4 (Abcam ab13840), lectin PNA (Invitrogen L21409), GCNA (DSHB 10D9G11), and vimentin (Biolegend 699301). Antibody specificity for immunofluorescent staining was validated by the manufacturers and further confirmed using knockout tissues and/or normal serum as negative controls. Fluorescent images were captured using a Zeiss LSM880 confocal microscope. Immunofluorescent signal intensity was quantified using ImageJ software with a freehand tool. TUNEL staining was performed using an *in situ* cell death detection kit (Roche 11684795910) according to the manufacturer’s protocol. TUNEL images collected from low-magnification whole testis sections were quantified by calculating the percentage of seminiferous tubules containing at least one TUNEL-positive cell relative to the total number of seminiferous tubules in the testis, *that is,* cell death index (65).

### Cell culture

*WT* and *SEL1L*^*−/−*^ HEK293T cells were generated as described previously (66). HEK293T and GC-4 cells were cultured in DMEM (Gibco) containing 10% FBS and 1% penicillin-streptomycin. For tunicamycin (Tuni, Tocris 3516) treatment, cells were treated with 2 μg/ml Tuni for 4 or 24 h, then collected for RNA or protein extraction.

### RNA sequencing

Testis RNA was isolated as previously described (12). Briefly, total RNA was extracted from the testis using RNA-Stat 60 (IsoTex Diagnostics), chloroform, followed by purification using a total RNA extraction kit (Omega Bio-Tek-R6834–02) with DNaseI digestion (Roche). RNA quality and concentration were examined using the Agilent tapeStation and AccuBlue Broad Range RNA Quantitation assay, respectively. RNA sequencing was performed by Admera Health. Briefly, paramagnetic beads coupled with oligo d(T)25 were combined with total RNA to isolate poly(A)+ transcripts based on NEBNext Poly(A) mRNA Magnetic Isolation Module manual (New England BioLabs Inc). Random priming and fragmentation were performed before first strand synthesis using Protoscript II Reverse Transcriptase. Library constructions were done according to the NEBNext Ultra II Directional RNA Library Prep Kit for Illumina (New England BioLabs Inc). Final libraries were assessed for quantity and quality with an average size of 430 bp. Equimolar pooling of libraries was performed based on QC values and sequenced on an Illumina Novaseq platform with 150PE reads (Illumina). A fold-change of two and adjusted *p*-value < 0.01 were set as cutoff for differential regulation. Pathway analysis was performed using the ClusterProfiler package in R.

### Statistics

Unpaired two-tailed Student’s *t* test was used for two-group analyses. Two-way ANOVA analysis with Tukey multiple comparison test or one-way ANOVA with Tukey *post hoc* test was used for multi-group analyses (GraphPad Prism). Data were presented as the mean ± SD; *p* < 0.05 was considered significant.

### Study approval

All animal procedures were approved by the Institutional Animal Care and Use Committee (IACUC) of University of Virginia and Wayne State University.

## Data availability

All study data are included in the article, supporting information, and supporting data file. High throughput RNA sequencing data of mouse testes have been deposited in the Gene Expression Omnibus (GEO) database under accession number GSE292552.

## Supporting information

This article contains supporting information.

## Conflict of interest

The authors declare that they have no conflicts of interest with the contents of this article.
