# Supplementary material for: The ER-associated degradation adaptor SEL1L is dispensable for ER homeostasis and the differentiation of spermatogenic cells
Source: J Biol Chem. 2025 May 22;301(7):110283. doi: 10.1016/j.jbc.2025.110283 (PMC12212276; doi:10.1016/j.jbc.2025.110283)
Supplement: Supporting information [file mmc1.pdf]

SUPPORTING INFORMATION:

The ER-associated Degradation Adaptor SEL1L is Dispensable for ER Homeostasis and the Differentiation of Spermatogenic Cells

Tushi et al.

Supplementary Figures 1-5

Supplementary Table 1

SUPPLEMENTARY FIGURES

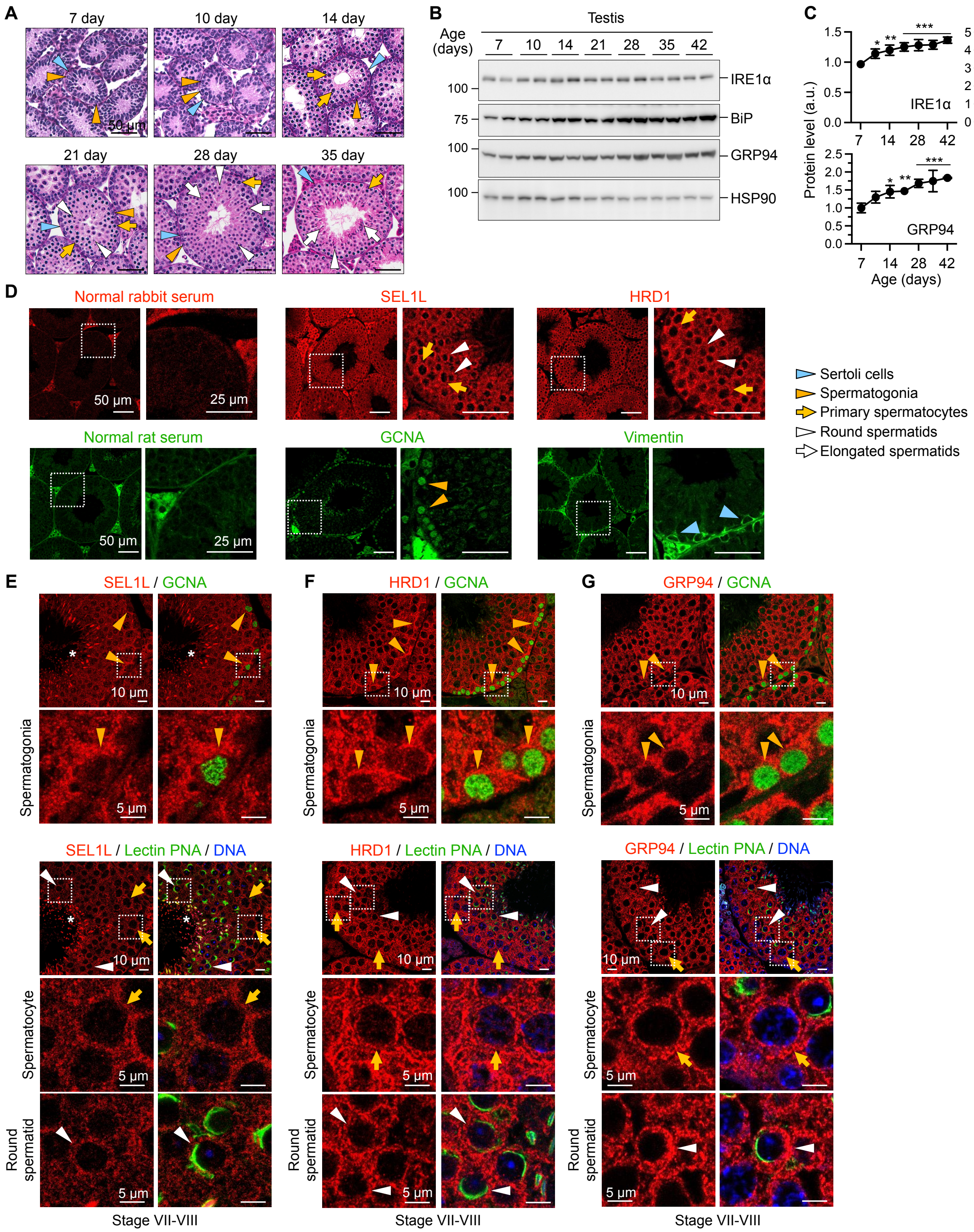

Figure S1. Expression of SEL1L and HRD1 in the testis. (A) Representative images of H&E stained testes sections from wild-type mice, showing various cell types during the first wave of the seminiferous epithelium cycle. N = 3 per cohort. (B-C) Western blot analysis of testes from wild-type mice at different ages, with quantitation normalized to HSP90 shown in (C). N = 4. Values, mean ± SD. *, p<0.05; **, p<0.01; ***, p<0.001 comparing different ages to 7 days by one-way ANOVA with Tukey multiple comparison test. (D-G) Immunofluorescent staining of testes from 6-week-old wild-type mice, showing negative controls using normal non-immunized rabbit or rat serum alongside corresponding primary antibodies (D), and the expression of SEL1L, HRD1, and GRP94 in various cell types as illustrated (E-G). GCNA labels spermatogonia; lectin PNA labels acrosomes in round and elongated spermatids. * indicates non-specific staining by the SEL1L antibody in spermatid flagella at stage VII-VIII. In D-G, insets of higher magnification are shown on the right or below. Representative data from n = 4.

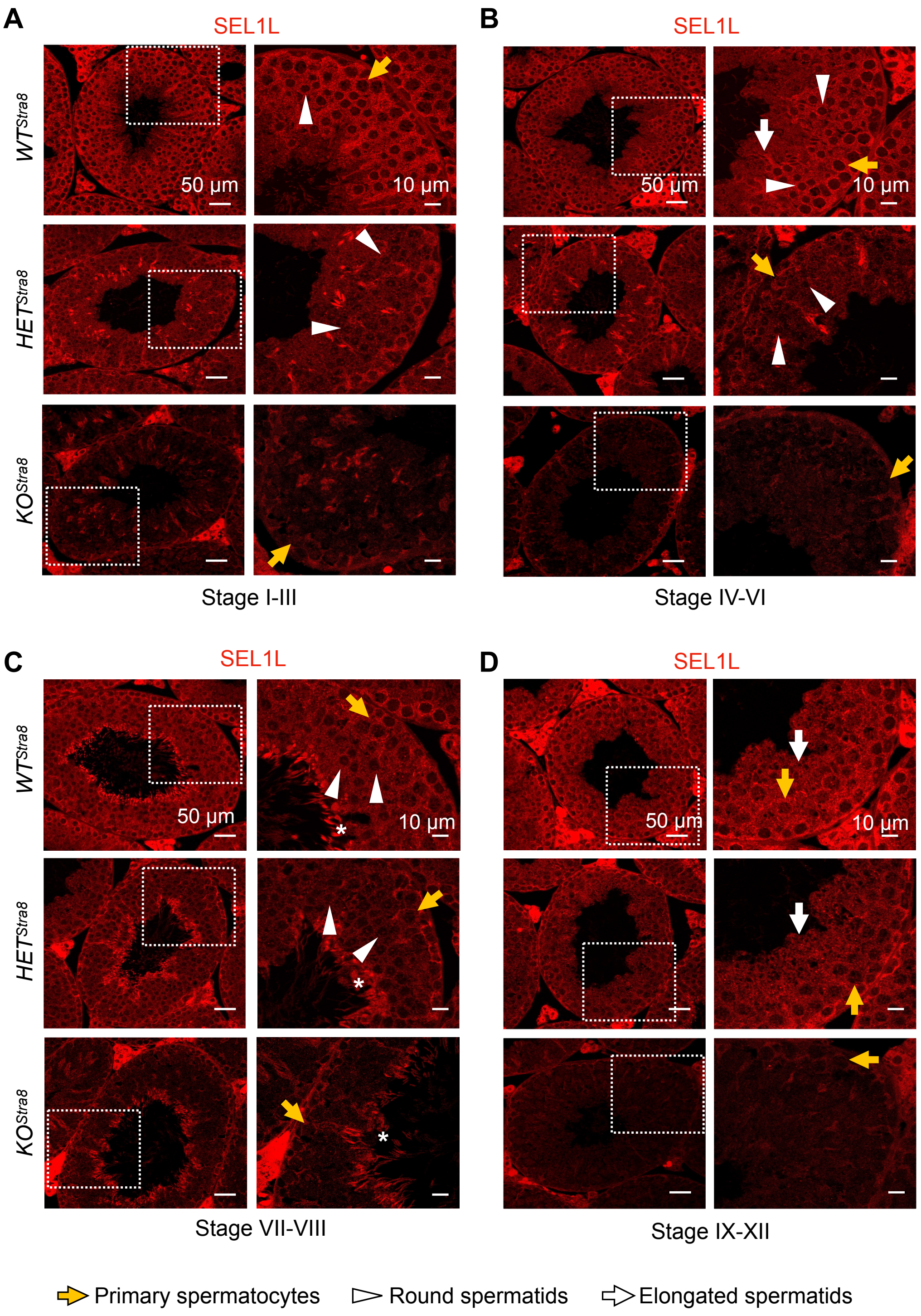

Figure S2. SEL1L is depleted in the spermatogenic cells of *KO*^{Stra8} mice. Representative immunofluorescent images of testes from 6-week-old *WT*^{Stra8}, *HET*^{Stra8}, and *KO*^{Stra8} littermates showing SEL1L ablation in spermatogenic cells at different stages of the seminiferous epithelium cycle. Insets of higher magnification are shown on the right. * indicates non-specific staining by the SEL1L antibody in spermatid flagella at stage VII-VIII. In *KO*^{Stra8} testes, round and elongated spermatids were difficult to identify due to the loss of SEL1L signal. Representative data from n = 4 mice per cohort.

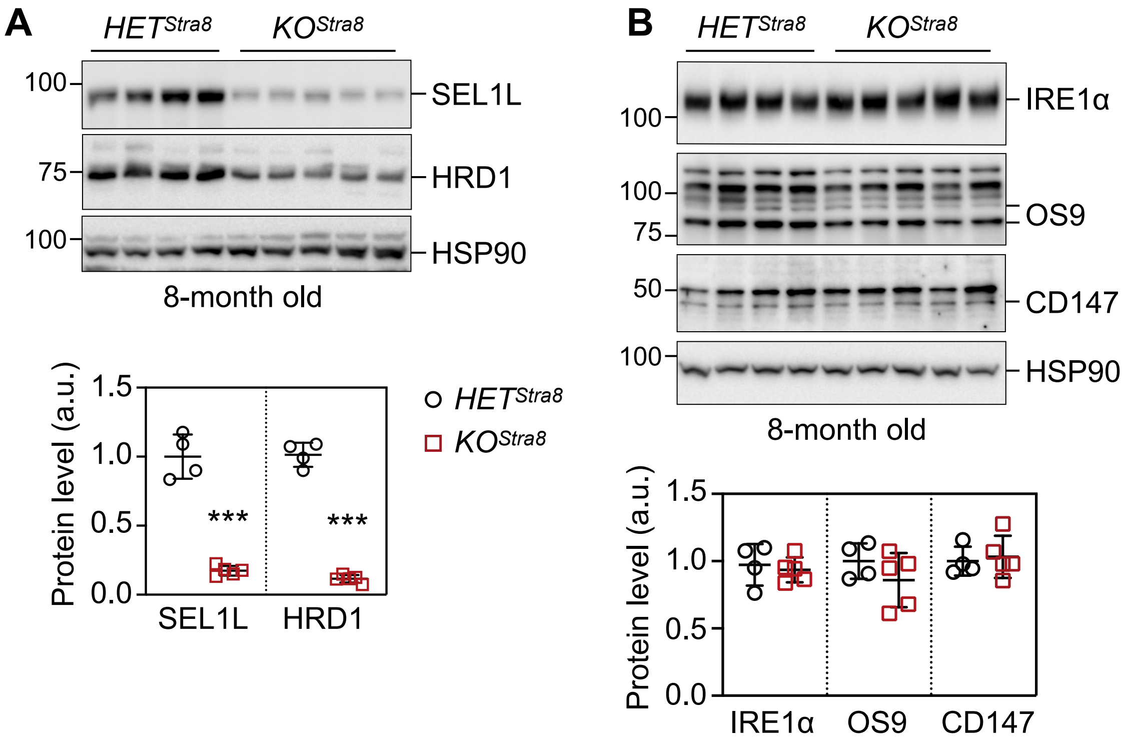

Figure S3. SEL1L is dispensable for ERAD activity in spermatogenic cells. Western blot analysis of testes from 8-month-old HET^{Stra8} and KO^{Stra8} littermates, with quantitation normalized to HSP90 shown below. Values, mean ± SD. ***, $p<0.001$ by two-tailed Student's t test.

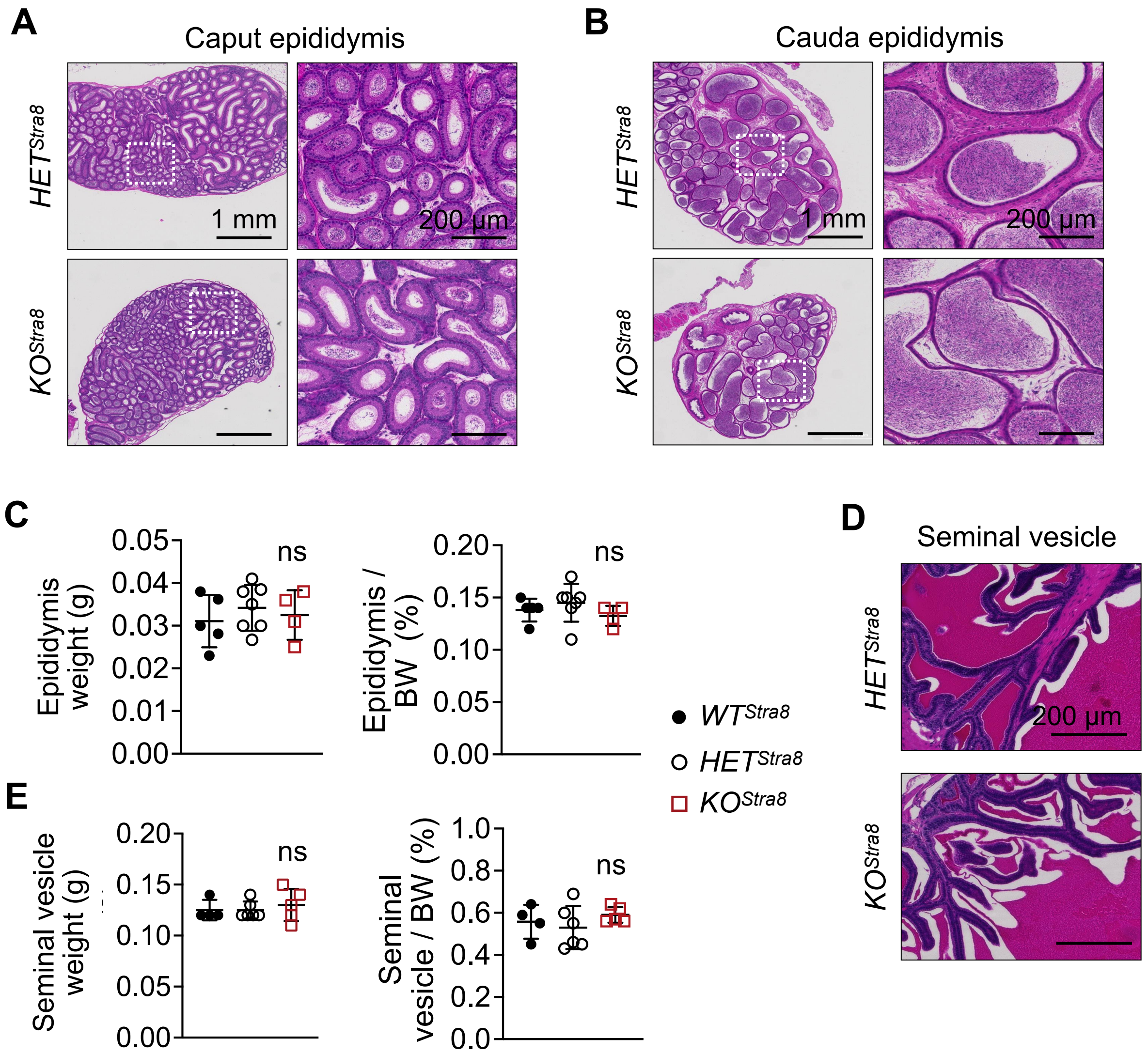

Figure S4. Epididymis and seminal vesicles in male germ cell-specific *Sel1L* knockout mice. (A-B) Representative H&E stained sections of caput and cauda epididymis from HET^{Stra8} and KO^{Stra8} littermates. Insets of higher magnification shown on the right. Representative images from n = 4 mice per cohort. (C) Weight and normalized weight of epididymis from WT^{Stra8}, HET^{Stra8}, and KO^{Stra8} littermates. N = 4 to 7. (D-E) H&E stained sections (E), and weight and normalized weight (E) of seminal vesicles from WT^{Stra8}, HET^{Stra8}, and KO^{Stra8} littermates. N = 4 to 6. Values, mean ± SD. Ns, not significant by one-way ANOVA with Tukey multiple comparison tests.

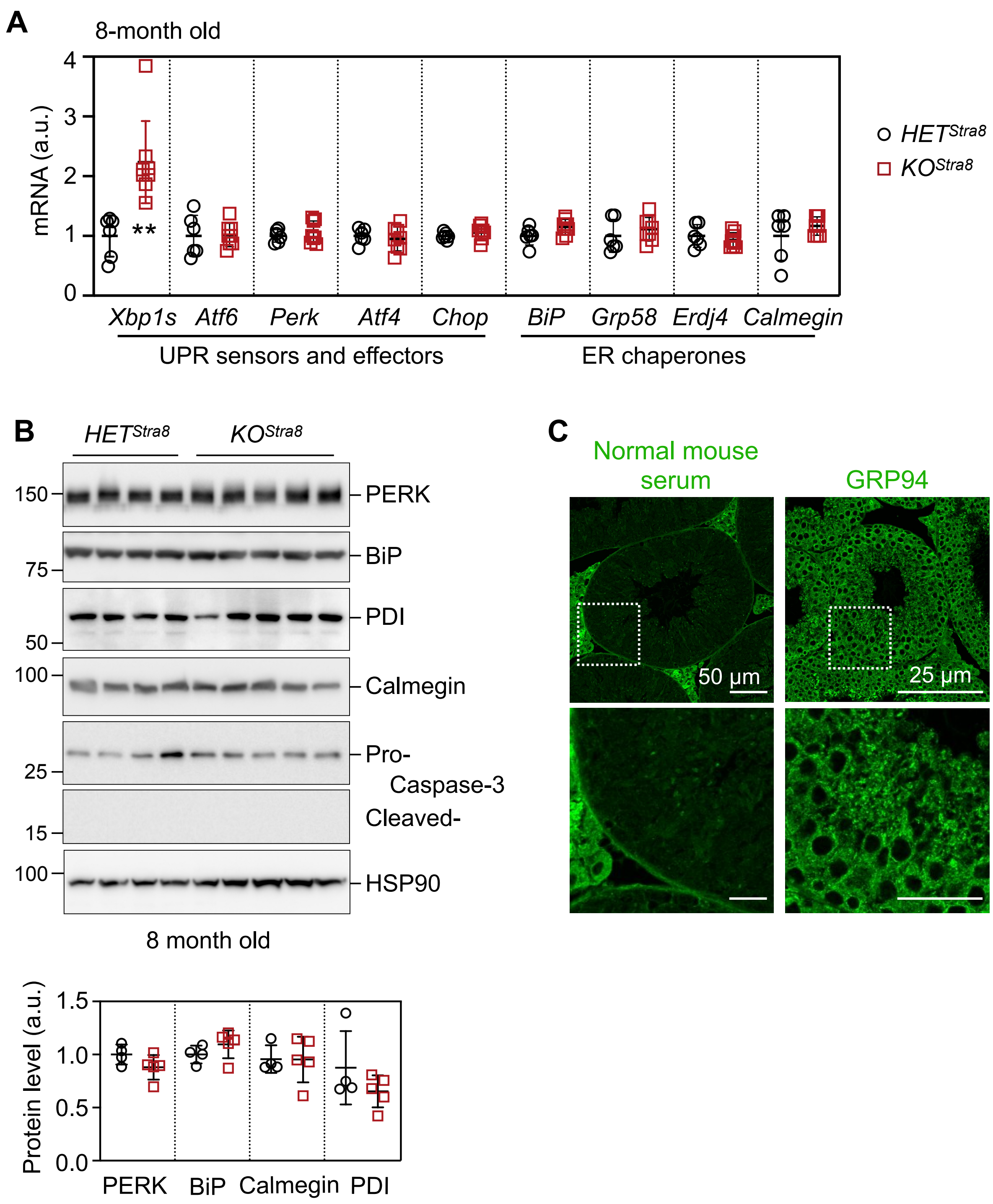

Figure S5. Lack of UPR after male germ cell-specific *Sel1L* ablation. (A) qPCR and (B) Western blot analyses of ER chaperones and UPR markers in testes from 8-month-old HET^{Stra8} and KO^{Stra8} littermates. In B, quantitation normalized to HSP90 shown on the right. Values, mean ± SD. **, p<0.01 by two-tailed Student's t test. (C) Immunofluorescent staining of GRP94 in testis sections from 6-week-old wild-type mice, with a negative control performed using normal non-immunized mouse serum. Insets of higher magnification are shown below.
